# PET imaging of mycobacterial infection: transforming the pipeline for tuberculosis drug development

**DOI:** 10.1038/s44303-025-00082-2

**Published:** 2025-05-28

**Authors:** Janke Kleynhans, Christiaan A. Gouws, Thomas Ebenhan

**Affiliations:** 1 Department of Pharmaceutical and Pharmacological Sciences, Radiopharmaceutical Research, Katholieke Universiteit Leuven, Leuven, Belgium; 2 Preclinical Imaging Facility, Nuclear Medicine Research Infrastructure NPC, Pretoria, South Africa; 3 Department of Nuclear Medicine, University of Pretoria, Pretoria, South Africa

**Keywords:** Biological techniques, Imaging, Positron-emission tomography

## Abstract

Improved PET/CT radiopharmaceuticals can better visualize and monitor tuberculosis and enable real-time pharmacological drug profiling in vivo. PET/CT imaging can therefore be used to study in animal models the changes in tissue pathology in tuberculosis infection, such as mycobacterial latency, tuberculoma formation, lung cavitation or calcification, and extrapulmonary disease. This Perspective aims to critically evaluate the current and future contribution and role of PET imaging in anti-tuberculosis drug development.

## Introduction

Mycobacteria are pathogens within the order of Actinobacteria; characterized as Gram-positive, aerobic, non-spore-forming bacilli. In addition to atypical, non-tuberculous mycobacteria, the two most recognized species of mycobacteria are *Mycobacterium leprae*, the causative pathogen for leprosy^1,2^, and *Mycobacterium tuberculosis* (MTb), the cause of tuberculosis (TB)^3^. Due to limited evidence in the literature supporting the usefulness of preclinical imaging in the development of novel therapies for leprosy, it will not be discussed further in this context. This may be due to the lack of systemic manifestations, at least on the preclinical level.

**Fig. 1:**
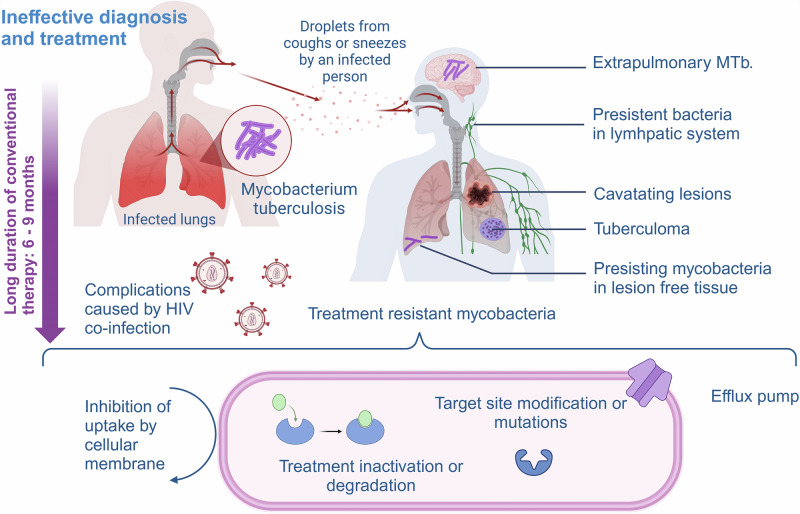
Interplay of complications during anti-mycobacterial therapy^11,15^. Figure created using a licensed version of Biorender.com.

Yet, TB is a lethal disease that has afflicted humanity for millennia, with DNA evidence tracing it back over 8000 years^4^. In 1882, Dr. Robert Koch identified the MTb bacillus, paving the way for treatments (antibiotics) and preventative measures such as the bacilli Calmette-Guerin vaccine^3^. Despite advances, MTb remains a global health threat, ranked as the second leading cause of death by infectious disease in 2022, after COVID-19. According to the WHO, 10.6 million people developed active TB, and 1.3 million died. MTb’s ability to cause latent infections in up to 1.7 billion individuals, which can reactivate, ensures a continuous reservoir of infection, making it highly transmissible within populations^5^. Once someone is latently infected, the chance of developing active TB is approximately 5% within the first two years^6^. Figure 1 illustrates the current disease profile and its complications with available therapeutic strategies. The treatment of MTb is intricate and prolonged, making the search for novel treatment regimens a high priority^7–9^. According to the U.S. Centers for Disease Control and Prevention (CDC) current clinical TB diagnosis involves a combination of medical history, physical examination, TB tests (i.e., skin tests and blood tests), chest X-rays, and bacteriologic examinations (including sputum smear microscopy, culture tests, and nucleic acid amplification tests). Follow-up care includes directly observed therapy to ensure medication adherence and regular monitoring through repeat imaging and lab tests to track treatment response and detect complications (CDC-Tuberculosis). To enhance the clinical translation success rate of new anti-TB therapies, the use of non-invasive nuclear imaging technologies such as Positron Emission Tomography (PET) is becoming increasingly crucial. When combined with Computed Tomography (CT), PET/CT enables the simultaneous visualization of both biochemical and anatomical information. These non-invasive techniques enable the longitudinal monitoring of TB disease processes in living subjects, providing valuable insights into the effectiveness of treatments^10^.

By using 2-deoxy-2-[^18^F]fluoro-D-glucose ([^18^F]FDG) in clinical TB research, researchers can explore the benefits of PET in monitoring mycobacterial activity in response to anti-MTb therapies. [^18^F]FDG-PET imaging detects metabolic activity in TB lesions, providing insights into disease progression and treatment efficacy. Visualizing MTb with new PET/CT imaging strategies is challenging due to the need for specificity, optimized pharmacokinetics, and minimal off-target effects. We herein highlight the growing importance of PET imaging in TB and describe the main challenges in developing new radiopharmaceuticals to monitor MTb-targeted therapies – which is crucial for evaluating new treatment regimens. We also compare TB animal models and several existing but underutilized PET tracers that could benefit clinicians, especially tracers that may offer more precise imaging of TB lesions, enhancing diagnosis and monitoring of treatments.

## The quest to conquer problems with TB diagnosis and treatment

MTb treatment is complicated due to multiple factors; treatment failure not only affects the host, but also contributes to the development and transmission of multidrug-resistant (MDR) and extensively drug-resistant (XDR)-TB, often causing larger cohort outbreaks of difficult-to-treat cases^11^. Aside from the socio-economic aspects burdening low-to-middle-income countries with the highest TB-related morbidity and mortality (Global Tuberculosis Report 2024), we herein focus on the pathogen-, host-, and treatment-related challenges. Concerning TB infections, MTb contributes unique **pathogen-related challenges** that complicate successful treatment. A heterogeneous TB infection often includes MTb bacteria at different physiological stages (replicating, dormant, reactivated), each with varying drug susceptibilities^12–14^. MTb bacilli can be found extracellularly and within immune cells - some reside in inaccessible lesions and drug-resistant niches, particularly in necrotic granulomas and cavities with poor drug penetration. Additionally, MTb can spread via the hematological and lymphatic systems, leading to extrapulmonary TB (EPTB). Additionally, MTb can develop drug resistance mechanisms through genetic alterations (Fig. 1) – changes leading to inhibited cell wall penetration of current anti-TB drugs, the activation of efflux pumps expelling these drugs, and target site modifications or mutations producing new enzymes capable of drug degradation^11,15^.

**Host-related challenges** significantly contribute to suboptimal treatment outcomes. HIV/AIDS co-infection poses the most pronounced challenge to anti-TB therapy success. A usual complication is the burden of undergoing both TB- and HIV therapy (so-called “pill burden”), often leading to non-adherence by patients^16^. In immune-suppressed HIV patients, the risk of developing active TB is twenty-to-thirty times higher compared to those without comorbidities^17^. HIV infection also increases the risk of reactivation of latent TB and rapid disease progression after infection. TB infection is reported as the single-largest cause of death among patients with AIDS^17–19^. Other comorbidities weakening the immune system include alcohol use disorders, diabetes, smoking, and malnutrition (6.3 TB determinants).

**Fig. 2:**
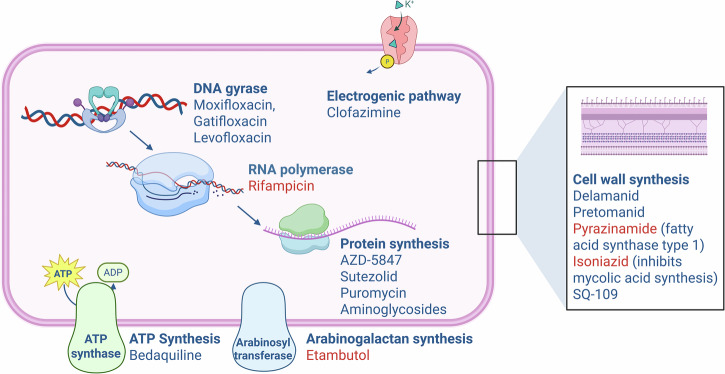
Available antibiotics for targeted MTb treatment and their mechanism of action. The figure includes standard therapy agents (red font color) and investigational drugs (blue font color) in advanced trials^7,8^. Figure created using a licensed version of Biorender.com.

Currently, TB is treated with a rather generic, 6-month multidrug regimen, causing **treatment-specific challenges**. Patient adherence remains a major concern due to the prolonged duration, requiring strict compliance to prevent relapse and drug resistance. Additionally, drug-related toxicities such as hepatotoxicity (isoniazid, rifampicin, pyrazinamide), gastrointestinal side effects, and optic neuropathy (ethambutol) can lead to treatment interruptions. Drug-drug interactions, particularly with rifampicin’s strong induction of hepatic enzymes, complicate co-treatment in patients with HIV or other comorbidities. These challenges highlight the need for shorter, less toxic, and more effective TB treatment regimens^20,21^. However, the onset of MDR-TB and XDR-TB further complicates treatment, requiring longer (more toxic) regimens. The lack of innovative antibiotic regimens hinders treatment of such MDR- and XDR-strains^11,15,21^. Therefore, developing new antibiotics, diagnostic biomarkers (including radiopharmaceuticals), and therapeutic strategies is crucial to address these challenges^22^. More focused therapies must simplify regimens to improve compliance — early diagnosis and detection of resistant strains may optimize treatments^23^. Nonetheless, recent reviews highlight progress on existing agents at revised dosages and introducing new agents like bedaquiline, pretomanid, and delamanid (Fig. 2)^24,25^.

The current one-size-fits-all approach is inadequate for managing TB effectively. To address the TB burden, we developed a task list {Box 1} which may significantly improve TB control and treatment efforts. For instance, vaccine development could be a powerful tool to stop the spread of MTb, especially in vulnerable HIV-positive populations or areas with poor socio-economic status^26^. It may offer a long-term, sustainable solution toward eradicating MTb and is the only effective weapon against the emergence and spread of MDR-TB and XDR-TB strains. However, while immunization has been key in reducing TB risk, its efficacy against adult pulmonary TB varies widely (0–80%)^27^. Challenges in developing new vaccines include an incomplete understanding of MTb’s pathogenicity, lack of correlates of protection, and absence of appropriate animal models for evaluation^28,29^.

**Diagnostic challenges** also remain. For example, molecular tests (e.g., Xpert MTB/RIF, Xpert Ultra, whole genome sequencing, line probe assays like GenoType, MTBDRplus, and other nucleic acid amplification tests), though useful, are still expensive and limited in certain TB samples. Culture-based methods take too long (2–8 weeks), complicating timely diagnosis and treatment. Recent reviews also highlight improvements made in point-of-care tests^24,25^.

Box 1 How to make inroads to curb the burden of TB

1. **Identification and development of biomarkers**: Biomarkers are needed to distinguish between patients with latent and active TB, and to evaluate treatment efficacy early on. This foundational step supports all subsequent efforts in TB management.
2. **Better identify LTBI patient subpopulations:** Using biomarkers and diagnostic tests, LTBI patients at higher risk of developing active TB can be identified, ensuring that they receive preventative treatment, while excluding low-risk individuals.
3. **Increase awareness of accurate and timely diagnosis**: Focusing on potent diagnostic methods helps to better identify and manage latent TB populations is essential for effective treatment and prevention strategies.
4. **Prioritize research for shorter, innovative treatment regimens or vaccines against TB**: Developing shorter, more effective treatments can improve patient compliance and outcomes. The implementation of improved diagnostics and biomarkers can expedite preclinical and clinical research that assesses drug or anti-TB vaccine efficacy.
5. **Identify gaps in healthcare systems**: Enhancing point-of-care tests, molecular diagnostics, and treatment capacity is vital for proactive TB surveillance and monitoring, ensuring that healthcare systems can effectively manage TB cases.
6. **Develop and secure access to innovative therapeutics worldwide**: Ensuring global access to treatments is crucial for effective TB management, particularly for latent TB infections (LTBI).
7. **Address socio-economic pressures**: Controlling treatment facility networks is essential to ensure a consistent supply of TB antibiotics/vaccines, addressing socio-economic barriers that hinder effective TB treatment and management.

## PET/CT imaging provides a uniform multi-species platform for preclinical anti-TB drug and vaccine development

**Fig. 3:**
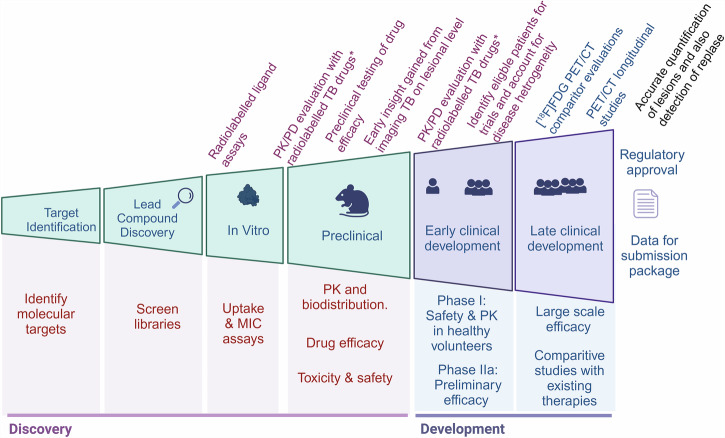
Implementation of PET/CT in the MTb drug development pipeline. * a radiolabeled (e.g., Fluorine-18 or Carbon-11) biosimilar version of the anti-TB drug will enable PET/CT imaging (non-invasive, longitudinal, quantitative investigations) for in vivo characterization. Figure created using a licensed version of Biorender.com.

The MTb antibiotic development pipeline relies on a robust foundation of preclinical animal models. Most animal models mimic only certain aspects of human TB disease, except for non-human primates, which closely resemble human TB. When studying TB in smaller animal models, a combination of models is often needed to fully characterize new therapies and vaccines because of the variation in which TB presents itself in different hosts, often mimicking only certain aspects of human TB disease. While preclinical evaluations can identify promising candidates, they cannot definitively predict clinical efficacy^28,30^. A preclinical efficacy study of a novel therapeutic should demonstrate either the potential to shorten current therapy regimens or increase efficacy against resistant MTb strains^9,20,21,30^. The more current focus within preclinical efficacy studies is evaluating the novel therapy’s efficacy against both active and non-active MTb, its activity against resistant strains of MTb, the possibility of development of resistance against the novel therapy, and pharmacokinetic (PK) and pharmacodynamic (PD) relationships that can affect therapeutic efficacy. Additionally, effectiveness against unique MTb lesion microenvironments, such as necrosis, cavities, and granulomas, is an auxiliary objective. For a drug to be effective, it must first reach its target, and all of these microenvironments affect the efficiency of this process. It is now accepted that the actual drug concentration in tissues of interest is directly correlated with therapeutic outcomes^20^. Hence, PK/PD-based dose selection of specific compartments of interest has become non-negotiable in the development of novel anti-MTb agents. Specifically in this area (Fig. 3), microPET/CT and PET/CT have unchallenged power.

While pharmacokinetics in blood can be assessed using conventional non-radioactive methods such as mass spectrometry or high-performance liquid chromatography (HPLC), these approaches are limited to measuring drug concentrations in blood or tissue homogenates at discrete time points. In contrast, microPET/CT and PET/CT offer the unique advantage of providing **real-time, whole-body** biodistribution and tissue penetration data in a non-invasive manner. This is particularly critical for TB drug development, where penetration into granulomas, lung lesions, and other infection sites cannot be accurately inferred from blood sampling alone. PET imaging enables dynamic tracking of drug distribution in living subjects, revealing spatial and temporal PK profiles that are otherwise inaccessible with traditional bioanalytical methods.

SPECT/CT is not commonly used in the drug development process for novel tuberculosis (TB) drugs due to its limited sensitivity and spatial resolution compared to other imaging modalities, such as PET/CT. While SPECT/CT can provide functional imaging, its lower resolution makes it less suitable for detecting early disease progression or subtle treatment effects. Additionally, the long half-life of most SPECT tracers limits their applicability for dynamic studies, and the availability of TB-specific radiotracers for SPECT is more restricted. Furthermore, radiolabelling novel antibiotics with SPECT-compatible radionuclides without altering their pharmacokinetics is challenging, whereas PET radiolabelling with short-lived isotopes like carbon-11 allows for minimal structural modification, preserving the drug’s biological behavior. As a result, PET imaging is generally preferred for assessing TB infection dynamics and treatment response.

PET/CT has found application in the evaluation of vaccine efficacy, in particular in macaques^31–37^, with smaller animal species remaining underutilized. [^18^F]FDG-PET/CT was proven to be effective at assessing disease establishment and dissemination, opening up the avenue to analyze how TB progression is influenced by novel vaccines. This is also a useful method to analyze the co-factors that could influence vaccine efficacy (e.g., correct dosing schedule, comparative efficacy, presence of co-morbities, etc.). Longitudinal assessment can provide a glimpse of the nuance of disease establishment/progression after vaccination, as well as assist in the determination of the duration of protection^31–37^.

Ensuring reproducible and translatable preclinical data for TB is crucial. Thus, the right combination of animal model, Mtb strain, and disease phenotype (or expression) must be aligned to address the research question accurately.

### Lack of diversity in selecting mycobacterial strains

In the reported studies, the diversity of MTb strains is limited, with H37Rv-, CDC1551-, and Erdman strains being commonly used^38^. This uniformity has enhanced repeatability and facilitated comparisons across different institutions; however, it also means that the genetic diversity of clinically isolated MTb strains is underrepresented. Consequently, data from drug-resistant Mtb strains is often missing. Because different strains can vary in virulence, immune evasion, and treatment response, validation without strain diversity may hinder the clinical translation of new MTb therapies^39^. To improve clinical translation, future studies should incorporate a range of MTb strains, including drug-resistant and geographically diverse clinical isolates, to assess treatment efficacy across different bacterial phenotypes. Additionally, using genetically diverse strains in animal models and patient-derived isolates in ex vivo studies can provide a more comprehensive evaluation of novel therapies.

Pulmonary TB is preferably established through aerosol infection, which closely mimics the natural respiratory route of human MTb transmission and provides acute infection in animal models. Other TB dissemination routes include (a) intrabronchial deposition, which best ensures correct pathogen dosing, (b) intraventricular injection relevant to illicit TB meningitis in animals, (c) intranasal droplet installation, and (d) intravenous MTb injection to directly introduce the pathogen systemically. More technically, aerosol-routed infection, for example, can be administered at ultra-low levels (1–3 CFUs; months-long incubation) or more conventional doses of 100–1000 CFUs, which may be given as a single dose or as repeated low doses. Aerosol infection or intrabronchial deposition is often used for imaging of acute pulmonary TB or conducting vaccine studies, while intrabronchial deposition is also chosen for evaluating granuloma development or disseminated TB disease^40^.

### For TB investigations, each animal model is different

**Fig. 4:**
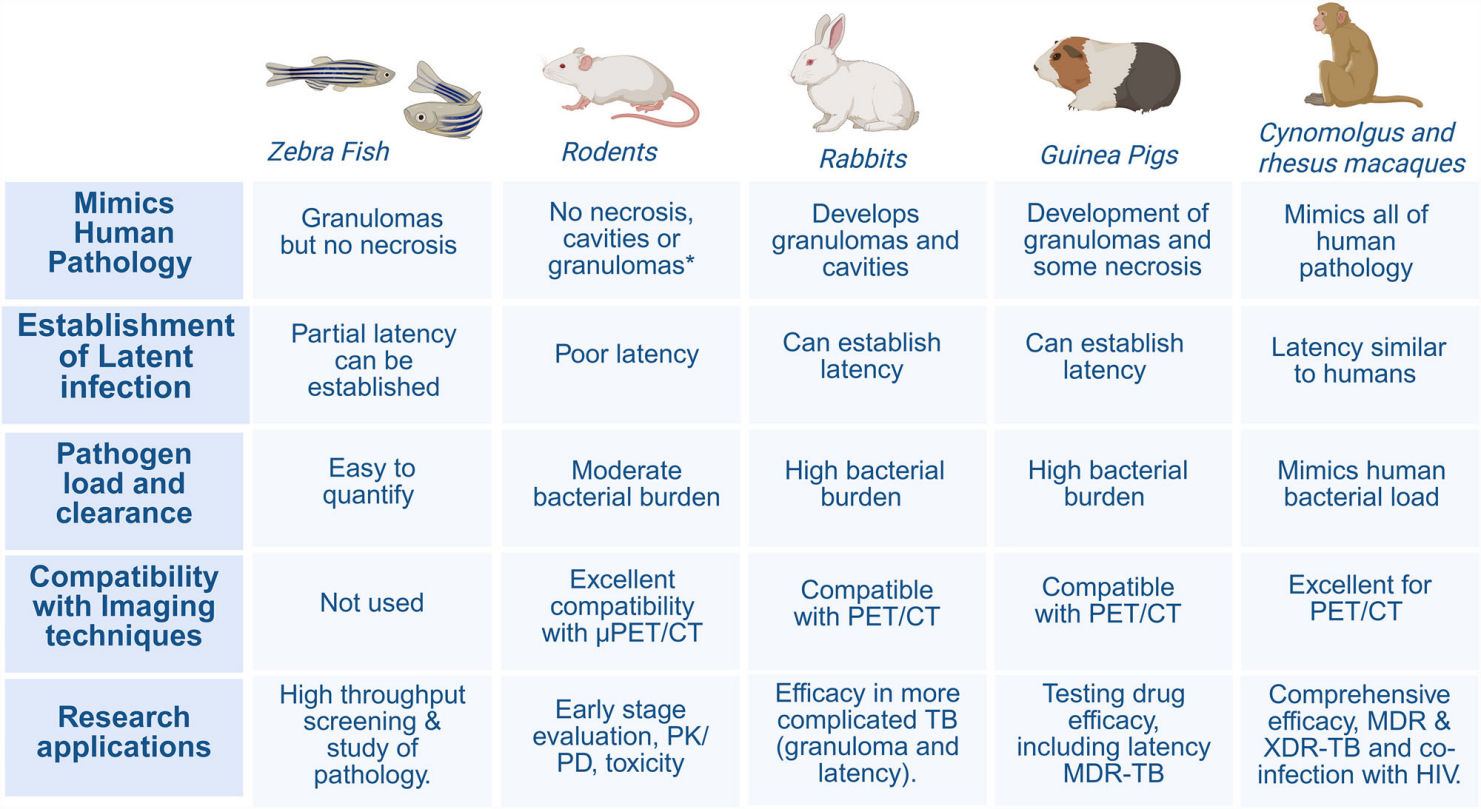
Comparing relevant animal models for evaluation of novel anti-MTb therapies^30^. *Human pathology is not replicated accurately, with the exception of specialized animal models like the C3HeB/FeJ ‘Kramnik’ mouse model. Figure created using a licensed version of Biorender.com.

Currently, mice are the most used preclinical species due to their cost-effectiveness and low compound dosage requirements. However, mice in general do not fully mimic human TB, lacking the three-dimensional structure and heterogeneity of human pulmonary TB lesions, next to the general differences already known between mouse and human regarding the anatomy and immune system. Mice in general are also relatively resistant to infection and show lower bacterial burdens, which can be detrimental to evaluating treatment duration or susceptibility to resistance in these animals^30,41^. However, specific sub-species of mice, for example, C3HeB/FeJ “Kramnik” mice, do develop TB granulomas in the lungs similar to humans. Rabbits and guinea pigs in general are more susceptible and manifest MTb infection that recapitulates the human pathology better, i.e., developing lung tissue necrosis and cavitation, which supports more in-depth drug characterization, and due to their small size, they can still be compatible with high-resolution microPET/CT imaging equipment. Non-human primates closely mimic the full spectrum of human MTb pathology^42,43^ but are limited by availability, cost, and ethical concerns^44,45^. Macaques are crucial for advanced evaluations of drug PK/PD and should preferably be used alongside smaller models for early-stage studies^20^. A summary of the key features for relevant animal models used in TB research and development is provided in Fig. 4; however, more comprehensive reviews have been published on this topic^30,41,46–48^.

### Several animal models of TB are suitable for PET/CT imaging

The introduction of microPET/CT in the preclinical development of MTb antibiotics promotes a more ethical and efficient research approach. This technology complies with the 3Rs (Replacement, Reduction, and Refinement) for ethical research^49^. Reduction is achieved through longitudinal imaging, allowing the same animal subjects to be studied over time, thus minimizing the animal number per study. Refinement is enhanced by PET/CT’s non-invasive nature, reducing the need for terminal procedures or repeated invasive sampling. This improves animal welfare, addressing pain and distress, especially in larger species like primates.

Macaques are the most commonly used animal models in TB-imaging studies due to their ability to monitor granuloma dynamics, immune system effects, and the progression from latent to active MTb^49–55^. Non-invasive imaging techniques like PET/CT are particularly powerful for these studies. PET/CT image-guided drug PK/PD data acquired from larger animal models are more translatable to humans. Conversely, C3HeB/FeJ “Kramnik” mice and rabbits are used for their cost-effectiveness and better ethical compliance, but at the cost of clinical relevance^46^. Most TB studies evaluate immune responses, vaccine efficacy, and the pharmacokinetics or efficacy of novel antibiotics. Primarily, [^18^F]FDG-PET is used, with a few studies using [^18^F]FMISO-PET to evaluate tissue hypoxia. The high reliance on [^18^F]FDG suggests a focus on inflammation in MTb pathology, potentially neglecting lesion-specific processes like hypoxia and necrosis that can’t be visualized with an [^18^F]FDG scan or PET/CT scan, since these tissues do not present with high metabolic activity.

We believe that using various radiopharmaceuticals during their preclinical PET/CT evaluations can accelerate the development of novel MTb therapies by eliminating non-compliant candidates early. The combination of high-throughput screening in TB-mice aligned with advanced PET/CT evaluations in primates is underutilized (perhaps underreported). Enhancing the bench-to-bedside translation success requires leveraging multiple animal models, each contributing unique strengths and weaknesses. Furthermore, advancing to high-throughput PET/CT studies is crucial for the scalable test of MTb antibiotics. Alas, longitudinal imaging capabilities are underutilized in PET/CT studies; simply presenting static images at a single time point risks neglecting or misrepresenting longitudinal treatment effects in the same animal cohort.

## The PET/CT imaging toolbox of TB goes beyond [^18^F]FDG

The ideal radiopharmaceutical supporting (pre-)clinical research of novel MTb therapy should demonstrate well-validated biological behavior in healthy subjects and in the disease process, most notably also in complicated disease. As such, [^18^F]FDG is highly metabolically active in the inflammatory process caused by TB infiltration^56^. This technique has been widely applied in non-human primate TB-models monitoring granuloma formation and selective granuloma dynamics^53,57,58^, tracking disease spread^53,59–62^, and guiding accurate tissue sampling during necropsy^63,64^. Disease severity and progression are monitored by quantitative measurements like total [^18^F]FDG-avidity, granuloma counts, or lymph node involvement to stage the disease^55,64,65^. [^18^F]FDG has been useful for evaluating treatment efficacy for novel MTb-directed antibiotics^59,63,66–68^, vaccines^31–37^ and host-directed therapies^69,70^, aided in biomarker development^71,72^, and in evaluating the risk of TB-reactivation^54,73^. Clinically, [^18^F]FDG-PET is valuable in the diagnosis and management of TB by detecting active disease and monitoring treatment response^74–78^. In more resource-limited settings with TB being most prevalent, PET imaging is often not readily accessible; however, despite this drawback, clinicians must be especially aware of its unique benefits and limitations. Therefore, we provide a fact sheet on [^18^F]FDG-PET/CT of clinical TB-imaging Table S1.

Aside from the plethora of [^18^F]FDG-PET investigations in TB, alternative metabolic PET tracers have been reported, mostly being studied side-by-side with [^18^F]FDG-PET/CT (Table 1). Note that non-metabolic radiopharmaceuticals and targets are discussed elsewhere in this manuscript.

**Table 1:** Readily available radiopharmaceuticals recommended for alternative metabolic PET/CT imaging of patients presenting with confirmed TB

Tracer	Cellular Function / Target	Relation to TB	Key Benefits (Limitations)
[^11^C]Choline^84^; [^18^F]FEC^85^	Increased biosynthesis of the cell (wall) envelope	Bacterial cell membrane biosynthesis	Improves specificity when utilized with [^18^F]FDG
[^11^C]Thymidine^86^ [^18^F]FLT^87^	DNA replication / elevated cell proliferation (TK-1 activity)	Bacterial proliferation and cellular division	Improves specificity when utilized with [^18^F]FDG
[^18^F]FMISO^88,89^	Tissue hypoxia	Tuberculoma hypoxia	Accurate differentiation and visualization of TB cavities (lower sensitivity than [^18^F]FDG)
[^68^Ga]Ga-citrate^90^	Serum transferrin cofactor / Krebs cycle intermediate	Bacterial iron-acquisition pathways	For better detection of EPTB (lower sensitivity than [^18^F]FDG)
[^11^C]Methionine^91^	Increased protein biosynthesis	Bacterial protein biosynthesis	For better detection of TB and EPTB
[^11^C]Acetate^92^	Fatty acid biosynthesis	Fatty acid biosynthesis is associated with tuberculoma development	Improves specificity when utilized with [^18^F]FDG

*[*^*18*^*F]FDG* 2-Fluoro-2[^18^F]-deoxyglucose, *EPTB* extrapulmonary tuberculosis, *DNA* deoxyribonucleic acid, *[*^*18*^*F]FLT* 3′-deoxy-3′-([^18^F])fluoro-thymidine, *[*^*18*^*F]FECh* [^18^F]fluoro-ethylcholine, *[*^*18*^*F]FMISO* [^18^F]fluoro-misonidazole, *[*^*18*^*F]FET* [^18^F]fluoro-ethyl-tyrosine.

A benefit for TB diagnosis (higher accuracy and anatomical localization of suspicious lesions), and detection of EPTB was reported, especially utilizing [^68^Ga]Ga-citrate-, [^18^F]FMISO-, [^11^C]choline- or [^18^F]FLT-PET. In a few clinical trials using receptor-specific imaging, patients with infectious (TB) diseases were enrolled as a control group to ascertain the specificity of new radiotracers for oncology. The researchers reported, for example, that [^68^Ga]Ga-PSMA-11-PET was unable to differentiate TB from pulmonary metastases; however, performing [^68^Ga]Ga-alfatide II-PET/CT imaging in TB patients had much higher specificity than [^18^F]FDG when distinguishing TB from Non-Small Cell Lung Cancer. Although PET investigations will most possibly not become part of the routine work-up for all patients diagnosed with TB, due to accessibility and resource limitations, the development of novel imaging strategies for TB pathophysiology is a lively area of research. The main aim is overcoming the [^18^F]FDG-PET/CT limitations toward TB, which provides a very inflammation-centric focus on the disease. Researchers require‘ more specific and sensitive tools for disease evaluation, most prominently during clinical trials of anti-MTb therapy and vaccines, but also during preclinical evaluations. Thus, researchers are aiming to develop pathogen-specific tracers that show uptake levels proportional to the bacterial burden. Such tracers could be valuable for patient stratification, monitoring response to treatment in complicated cases, and accelerating the development of novel therapeutics. Patient stratification is crucial because TB presents a spectrum of disease phenotypes, and grouping patients with different phenotypes in clinical trials may produce inconsistent results regarding treatment efficacy. A non-exhaustive list of new tracers, including their potential as drug efficacy PET-biomarkers is summarized in Table 2.

**Table 2:** Overview (non-exhaustive) and development status of novel PET radiotracers for TB imaging

Tracer name	MoA / cellular target	Status	Key notes	Role as anti-MTb drug efficacy PET-biomarker
[^68^Ga]Ga-DN3^93^	Tuberculoma hypoxia	CS	Visualization of hypoxia in a few patients with active TB (low sensitivity)	n.d.
[^64^Cu]Cu-ATSM^94^	Tuberculoma hypoxia	PC	Appropriate imaging of the necrotic tuberculoma microenvironment	+
[^18^F]Sodium fluoride^95^	Tissue micro-calcification caused by MTb tissue pathology	PC	Accurate detection of tissue pathology due to Mtb manifestation	++
[^68^Ga]Ga-FAPI(04)^96–98^	Inhibits fibroblast-activated protein in tuberculoma cells	CS	Sensitive visualization of pulmonary TB lesion (not TB specific)	n.d.
[^18^F]FDT^99^	MTb-specific cell wall uptake via the trehalose pathway	PC	Accurate visualization of pulmonary TB lesion (sensitivity equal to [^18^F]FDG-PET)	++
[^68^Ga]Ga-UBI_29-41_^100^	Antimicrobial peptide for bacteria-specific targeting of the cell wall	PC / CS	Accurate visualization of pulmonary TB and EPTB	n.d.
[^64^Cu]Cu-LL2PA^101^	Peptidomimetic ligand targeting -VLA-4 (α4β1) -expressing cells in tuberculomas	PC	Improved specificity for TB, tracer uptake driven by Mφ and T-cells	_
[^64^Cu]Cu-cFLFLF^102,103^	Modified peptidetargeting formyl peptide receptor 1 expressed on tuberculoma cells	PC	Accurate imaging of MTb-associated inflammation, uptake driven by neutrophils (appropriate sensitivity)	n.d.
[^18^F]ICMT-11^104^	Isatin-containing tracer, specific to caspase-3/7 expressed on Mφ	PC	Accurate PET imaging of caspase-3/7-specific activity during cell apoptosis (cisplatin-induced) in tuberculoma	++
[^11^C]JNJ-28312141^105^	Binds to colony-stimulating factor 1 receptor expressed on Mφ -crucial during TB infection	PC	Effectively delineating granulomatous foci of TB	+
[^124^I]iodo-DPA-713^106,107^	Binds to TSPO expressed on Mφ during TB infection	ECI /PC	Accurate, sensitive, and more specific imaging of MTb-associated inflammation (compared to FDG)	++
[^68^Ga]Ga-puromycin^108^	Measures increased protein biosynthesis	PC	Accurate visualization of BCG-based lung pathology (low sensitivity)	+
2-[^18^F]INH^109^	MTb-specific pro**-**drug (blocks type II fatty acid synthase)	PC	Accurate and sensitive visualization of pulmonary TB lesion (slow PK, moderate sensitivity)	_
5-[^18^F]F-PZA^110^	MTb-specific drug derivative (activated by pyrazinamidase)	PC	Accurate visualization of pulmonary TB (enzyme inhibitor with low sensitivity)	_
[^18^F]Pretomanid^83^	Inhibiting mycolic acid biosynthesis	PC/ FIH	Favorably low pulmonary uptake and radiation dosimetry FIH; high uptake in a TB meningitis model (mycobacteria-selective)	++
[^11^C]PABA^111^ 2-[^18^F]F-PABA^112^	Anti-tubercular enzymatic antifolate action	PC/ FIH	MoA prevalent in MTb. Favorably low pulmonary uptake and radiation dosimetry FIH. Not mycobacteria-specific	++
[^18^F-/^124/125^I](iodo)-FIAU^86,113,114^	Imaging of bacterial TK-1 expression via metabolic cell entrapment	PC/FIH	Favorably low pulmonary uptake and radiation dosimetry FIH; appropriate in vivo sensitivity (not bacteria-selective)	++

*MTb* Mycobacterium tuberculosis, *EPTB* extrapulmonary tuberculosis, *PC* preclinical investigation, *FIH* first-in human, *ECI* exploratory clinical investigation, *CS* series of clinical cases, *MoA* mechanism of action, *VLA* very-late antigen, *DN3* DOTA-nitroimidazole, *2-[*^*18*^*F]INH* 2-[^18^F]fluoroisonicotinic acid hydrazide, *[*^*18*^*F]FDT* 2-[^18^F]fluoro-2-deoxy trehalose; *DPA-713* N,N-diethyl-2-(4-methoxyphenyl)-5,7-dimethyl- pyrazolo[1,5-a]pyrimidine-3-acetamide, *TSPO* translocator protein, *Mφ* macrophages, *PABA* para-aminobenzoic acid, *FIAU* 2′deoxy-2′-fluoro-β-D-arabinofuranosyl-5-iodouracil.

Interestingly, unlike PET imaging for cancer, TB imaging literature/ has primarily focused on small-sized radiolabeled molecules. Larger molecules, such as radiolabeled antibodies and their fragments, functional proteins, aptamers, or bio-nanoparticles, have not been explored for PET/CT imaging of TB. Additionally, despite radiolabeled blood elements being the gold standard for imaging peripheral infections, studies visualizing EPTB foci by way of trafficking Zirconium-89-labeled leukocytes have not been reported either. This gap highlights a significant opportunity for advancing TB imaging techniques and improving treatment monitoring capabilities.

## The potential of PET/CT imaging during the development of a new TB therapy agent

**Fig. 5:**
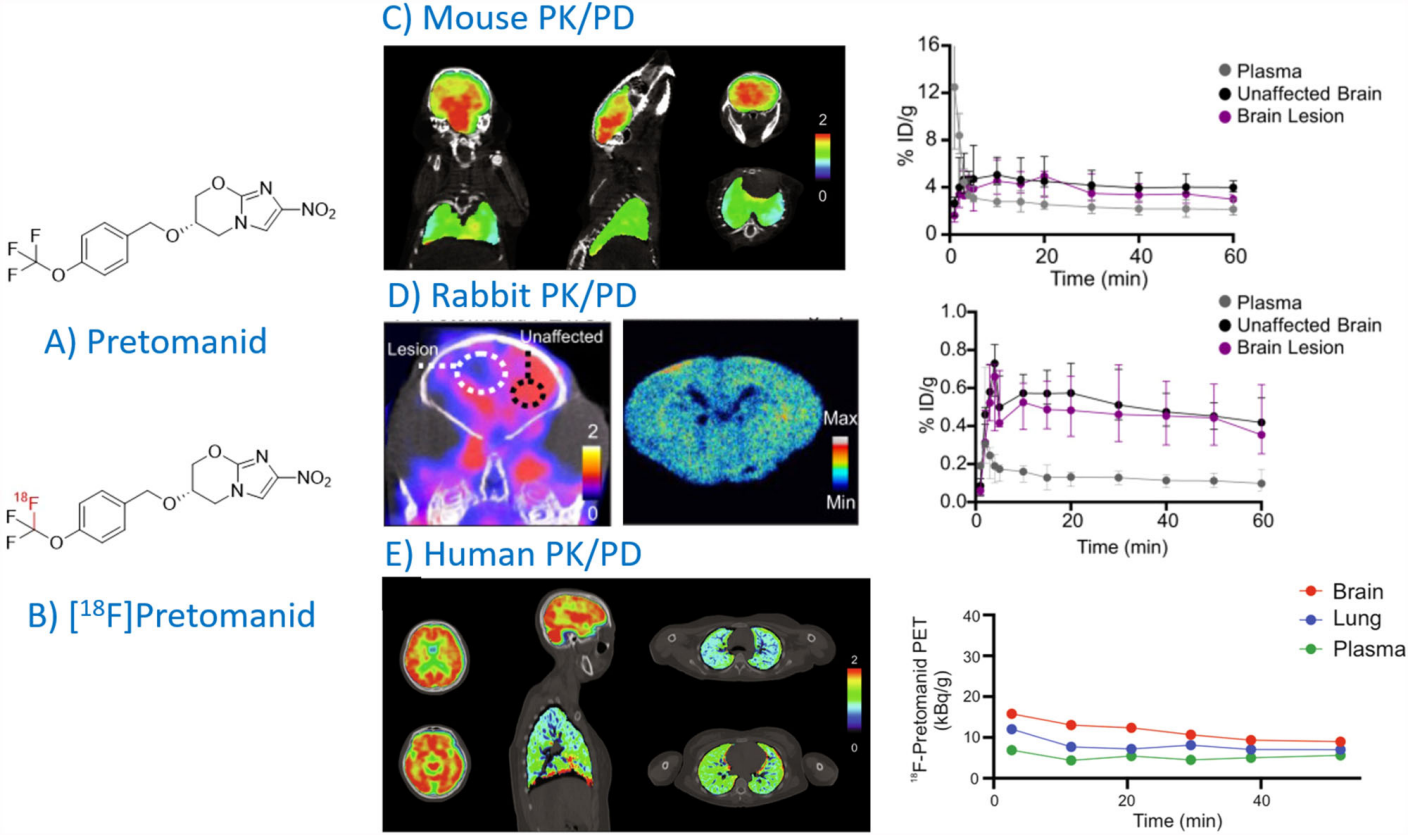
An example of the pharmacokinetic evaluation of Pretomanid using the biosimilar radioactive derivative [^18^F]Pretomanid and PET/CT imaging^25,83^. The structure of the unradioactive (**A**) and fluorine-18 labelled (**B**) compound is provided. Biological evaluation in a mouse model (**C**), rabbit model (**D**) and human subjects (**E**) was performed.

We herein emphasized the importance of incorporating PET/CT techniques in developing new anti-MTb drugs. Investigational drugs have been radiolabeled for real-time in vivo PET imaging, e.g., [^18^F]Linezolid, [^76^Br]Bedaquiline, and [^11^C]Rifampin^25,79,80^. To further illustrate the importance of incorporating nuclear imaging technologies, we propose recent developments of Pretomanid for combination therapies targeting drug-resistant strains of MTb - as an example. Pretomanid inhibits mycolic acid biosynthesis and shows uptake under anaerobic and hypoxic conditions^81^. It is unclear if Pretomanid can serve as a key drug in anti-MTb regimens or merely as an add-on to prevent resistance. However, it has proven effective in reducing treatment duration and preventing bedaquiline-resistant MTb strains. Although no serious toxicity has been reported, Pretomanid’s safety profile needs further investigation^82^. For Pretomanid, fluorine-18 was the radioisotope of choice as F-atoms form part of the molecule (compare Fig. 5A, B). It is crucial that the radio-analog is chemically (as) identical (as possible) to the non-radioactive antibiotic and behaves similarly. However, for accurate imaging results, sufficient proteolytic stability of the radiolabeled antibiotic in vivo is also desired. In developing Pretomanid, PET/CT was used to define compartment-specific pharmacokinetic data accurately^83^ (Fig. 5C–E). Traditional dosing regimens often rely on plasma concentrations, overlooking the importance of antibiotic distribution in critical compartments like the brain, or even in the infected tissue microenvironment. This can lead to sub-therapeutic levels of antibiotics in the tissues where the pathogen resides, which may result in pathogen survival and development of antibiotic resistance. Hence, radiolabelling new antibiotics like Pretomanid allows for three-dimensional and precise visualization of antibiotic exposure at infected tissue sites with PET/CT, and provides segmented (or dynamic) whole-body drug concentration profiles at multiple timepoints in an individual subject.

**Fig. 6:**
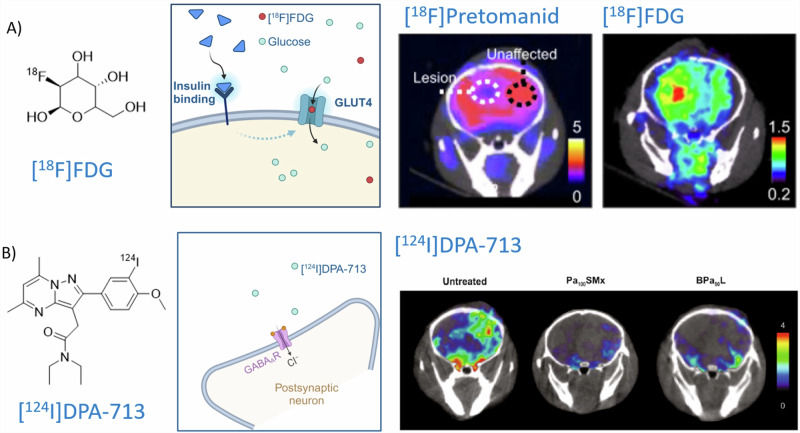
PET imaging further characterizing cerebral Pretomanid-based effects, using selected established radiopharmaceuticals. The figure demonstrates the distribution of **A** [^18^F]FDG and **B** [^124^I]*iodo*-DPA-713. Figure content was adopted from a previous publication^25^. Parts of the figure were created using a licensed version of Biorender.com.

Co-imaging using [^18^F]FDG (Fig. 6A) highlighted metabolic TB lesion activity (circled) and activated immune cells non-invasively, which can be correlated to decreasing inflammation and bacterial burden as a response to therapy^25^. In this study, the lack of [^18^F]Pretomanid uptake is evident; however, [^18^F]FDG clearly highlights the inflammation associated with MTb manifestation, indicating differential penetration of glucose versus an antibiotic such as Pretomanid, hinting at underlying molecular mechanisms that exclude antibiotic penetration of the lesion, which is still permissive to food sources like glucose. This setting is suitable for follow-up therapeutic efficacy over time in a quantitative manner. In addition, cerebral [^124^I]*iodo*-DPA-713-PET/CT images were reconstructed for representative (TB-infected) mice from different treatment regimens and untreated mice (Fig. 6B). [^124^I]Iodo-DPA-713 is a radiotracer used to detect and visualize macrophage-associated inflammation by measuring the activity of the 18-kDa translocator protein (TSPO), which is valuable for studying chronic inflammatory diseases. The reduction in MTb burden after therapy correlated with the decrease in in vivo PET signal intensity representing activated microglia and macrophages. These results showed that PET imaging allows for precise localization of infected tissues, quantification of disease activity, and measurement of therapeutic penetration and its efficacy, sparking confidence about the future role of PET imaging of mycobacteria, although only a selected few centers worldwide have the laboratory infrastructure to perform such research due to strict radiation and bio-contaminant regulation.

## Summary and outlook

PET imaging holds great promise for transforming TB diagnosis, treatment, and drug development, paving the way for more effective and personalized approaches to combating this challenging disease. New PET imaging techniques are set to revolutionize TB research, with emerging PET radiopharmaceuticals playing a crucial role in the development of new anti-TB drugs and vaccines. By illuminating the complex interactions between MTb and the host immune system, PET imaging will deepen our understanding of TB, potentially bridging the gap to novel therapeutic strategies and improved disease management. With PET imaging offering personalized care, it can help tailor treatments to patients with complicated MTb infections. However, for this to be realized, it is essential for PET/CT technology to become more accessible.

## Supplementary information

Supplementary Information

## Data Availability

No datasets were generated or analyzed during the current study.

## Abbreviations

[^18^F]FDG: 2-deoxy-2-[^18^F]Fluoro-D-glucose
[^18^F]FDT: 2-[18F]fluoro-2-deoxy trehalose
[^18^F]FECh: [18F]fluoro-ethylcholine
[^18^F]FET: [18F]fluoro-ethyl-tyrosine
[^18^F]FLT: 3′-deoxy-3′-([18F])fluoro-thymidine
[^18^F]MISO: [^18^F]Fluoro-misonidazole
2-[^18^F]INH: 2-[18F]fluoroisonicotinic acid hydrazide
3Rs: Replacement, Reduction, Refinement
CT: Computed Tomography
DNA: Deoxyribonucleic Acid
DPA-713: N,N-diethyl-2-(4-methoxyphenyl)-5,7-dimethyl-pyrazolo[1,5-a]pyrimidine-3-acetamide
EPTB: Extra-pulmonary tuberculosis
FIAU: 2′deoxy-2′-fluoro-β-D-arabinofuranosyl-5-iodouracil
HIV: Human Immunodeficiency Syndrome
LTBI: Latent tuberculosis infections
MDR-TB: Multi-Drug-Resistant Tuberculosis
MIC: Minimum Inhibitory Concentrations
MoA: Mechanism of Action
MTb: Mycobacterium tuberculosis
PABA: Para-aminobenzoic acid
PET: Positron Emission Tomography
PK/PD: Pharmacokinetics/Pharmacodynamics
TB: Tuberculosis
TSPO: Translocator protein
XDR-TB: Extensively drug-resistant tuberculosis
