# Supplementary material for: PET imaging of mycobacterial infection: transforming the pipeline for tuberculosis drug development
Source: Npj Imaging. 2025 May 28;3:22. doi: 10.1038/s44303-025-00082-2 (PMC12441125; doi:10.1038/s44303-025-00082-2)
Supplement: Supplementary file 1 — Supplementary Information [file 44303_2025_82_MOESM1_ESM.pdf]

Supplementary information to:

PET Imaging of Mycobacterial Infection: Transforming the Pipeline for Tuberculosis Drug Development

Janke Kleynhans[1], Christiaan A Gouws[2], Thomas Ebenhan[2,3*]

[1] Department of Pharmaceutical and Pharmacological Sciences, Radiopharmaceutical Research, Katholieke Universiteit Leuven, Leuven, Belgium

[2] Preclinical Imaging Facility, Nuclear Medicine Research Infrastructure NPC, Pretoria, South Africa

[3] Department of Nuclear Medicine, University of Pretoria, Pretoria, South Africa

*Corresponding author: thomas.ebenhan@up.ac.za

Table S1: Key benefits and limitations of [^{18}F]FDG-PET/CT in clinical imaging of tuberculosis (*to be moved in a Supplement data file for publication*).

Feature/Limitation	Description
**Detection Accuracy**	High accuracy and sensitivity for detecting active tubercular lesions, even when X-rays and sputum tests are negative. Helps to identify subjects from specific patient populations.
**Whole Body Imaging**	Effective in detecting extrapulmonary tuberculosis (diagnostic challenge for other methods) due to holistic image acquisition.
**Functional and Anatomical Imaging**	Combines metabolic activity data with detailed anatomical images for better localization and characterization of tubercular lesions. Supports early-stage development of new radiopharmaceuticals.
**Soft Tissue Assessment**	Excellent for assessing metabolic activity in soft tissues, though limited by CT scan resolution for localisation of small or dispersed lesions. PET/MRI could be a future solution.
**Disease Extent Assessment**	Provides quantitative data on lesion metabolic activity, crucial for randomization for treatment planning and monitoring response (high sensitivity required).
**Lack of Specificity**	Potential for false positives due to high [^{18}F]FDG uptake in other conditions like malignancies and inflammatory processes, complicating differentiation from active tuberculosis.
